# What is the Quality of Life of Transtibial Amputees in Brunei Darussalam?

**DOI:** 10.5704/MOJ.2007.009

**Published:** 2020-07

**Authors:** SS Ng, L Naing, FI Idris, K Pande

**Affiliations:** 1 PAPRSB Institute of Health Sciences, Universiti Brunei Darussalam, Gadong, Brunei Darussalam; 2 Department of Orthopaedics and Traumatology, Raja Isteri Pengiran Anak Saleha Hospital, Bandar Seri Begawan, Brunei Darussalam

**Keywords:** quality of life, transtibial amputation, below knee amputation, rand 36-item health survey, houghton scale

## Abstract

**Introduction:**

Lower limb amputations have a profound impact on the quality of life (QoL) of the patients. This study was done to assess the QoL of patients with transtibial (below-the-knee) amputations (TTA) and transtibial amputees fitted with prosthesis.

**Material and Methods:**

A case-control study of patients who had undergone TTA from 2015 to 2018 was conducted in Raja Isteri Pengiran Anak Saleha Hospital (RIPAS). Complete data was available for 30 subjects and it was compared with 30 diabetic, non-amputee patients matched for age and gender. QoL was assessed using the RAND 36-Item Health Survey (SF-36) and the functional outcome of prosthesis-fitted transtibial amputees was assessed using the Houghton Scale.

**Results:**

Almost all cases of TTA were a result of vascular problems related to diabetes and chronic renal disease (n=29; 97%). Eighteen (60%) participants were fitted with prosthesis and 15 (50%) reported having phantom pain. QoL of participants was found to be significantly lower than that of age and sex-matched diabetic non-amputees with regards to physical functioning, role limitation due to physical health, emotional well-being, social functioning, and bodily pain. The mean Houghton Score for participants fitted with prosthesis was 4.89 (SD= 2.83) suggesting low functional outcome.

**Conclusion:**

TTA has a negative impact on the QoL of patients, especially in terms of functionality. The availability of prosthesis does not significantly improve the quality of life except in the physical functioning domain. Emotional well-being should be emphasised more in the rehabilitation process as this study found poor emotional well-being among participants.

## Introduction

Amputation is a surgical procedure that causes permanent disability and major physical as well as psychological alterations, which are felt more by the major extremity amputees^1^. Peripheral vascular disease, physical trauma and neoplasms, especially osteosarcoma, are the main reasons leading to lower limb amputation^2^. Non-healing diabetic ulcers in the lower extremities can progress to cellulitis, abscesses, osteomyelitis, gangrene, and amputation may be necessary to prevent the spread of infection from the necrotic tissue^3^.

Lower limb amputation is more common than upper limb amputation and is mostly due to vascular problems from diabetes and chronic kidney disease. In Malaysia, a study of 37 lower limb amputees showed that 86% were unilateral amputees, 54.8% had below knee amputations and most were diabetic^4^. Similarly, a study of 81 patients in Brunei Darussalam had confirmed that diabetes mellitus was the leading cause of lower limb amputation while TTA was the most common procedure performed^5^.

The World Health Organization defines quality of life as ‘an individual’s perception of his/her position in circumstances of the culture and values in which he or she lives and with respect to his/her goals, expectations, principles and concerns’^6^. Quality of Life (QoL) is recognised as a major predictor of rehabilitation programs and has mainly been used to compare the effectiveness of these programs or to compare amputees with diseased or normal population. QoL also reflects the holistic health status of a patient as it not only includes physical health, but also mental health^7^.

Previous studies have confirmed lower QoL in lower limb amputees compared to the general population and in those with major amputations compared to minor amputations^7, 8^. Currently, there is no data available on the QoL in TTA patients in Brunei Darussalam. This study investigated the quality of life of lower limb amputees specifically on patients who had TTA, by comparing the quality of life between TTA patients and age and sex-matched diabetic non-amputees; those fitted with prosthesis and those without.

## Materials and Methods

Data of patients who underwent TTA from 1st January 2015 to 31st December 2018 was obtained from the hospital records via the Brunei Health Information Management System. The inclusion criteria included patients aged 18 to 70 years old who were citizens or permanent residents of Brunei Darussalam, had completed primary wound healing and were undergoing or had undergone rehabilitation under the Rehabilitation Unit, RIPAS Hospital, the main tertiary hospital in the country. Those who had impeded gait patterns due to open wounds, traumatic brain injury, spinal cord injury, neurological, and psychological or mental disorders were excluded from this study.

A total of 107 TTA cases from 2015 to 2018 were retrieved from the hospital’s database, of which 59 patients survived up to the point of our study. Forty-nine of them met our study’s inclusion criteria, but only 30 of the 49 patients could be contacted and consented to participate in this study.

Data of the TTA patients that were collected included their sociodemographic characteristics i.e. age, gender, ethnicity, marital status, education level, employment status, comorbidities (diabetes, hypertension, hyperlipidemia, renal disease); amputation characteristics - causes (diabetes or vascular, trauma), duration of amputation, amputation side (left or right), presence of prosthesis, any stump or phantom pain and stage of wound healing.

Participants completed the RAND 36-Item Health Survey version 2 (SF-36) and the Houghton Scale. The SF-36 is one of the most widely used health-related quality of life validated instrument, comprising of 36 items that assess eight health concepts: physical functioning, role limitations caused by physical health problems, role limitations caused by emotional problems, social functioning, emotional well-being, energy/fatigue, pain, and general health perceptions, where a high score defines a more favourable health state^9^. The Brunei-Malay version SF-36 version 2 was used which was adopted from the Malaysian-Malay SF-26 v2 and has been validated in a separate study of patients with chronic renal disease^10^.

The Houghton Scale consists of four questions that reflect an individual’s perception of prosthetic use in lower limb amputations^11^. Results are reported as a total score out of 12, with higher scores indicating greater performance and greater comfort. A score ≥ 9 on the Houghton scale is indicative of satisfactory rehabilitation. A score between 6 and 8 is indicative of mobility on the prosthesis around the house and limited community walking ability. Scores ≤ 5 corresponds to limited household walking ability^11^. The Houghton Scale was translated into the Malay language by the researcher and pre-tested among a group of 10 peers of the researcher. Feedback on the questionnaires was received and appropriate changes were made to improve the language and understanding. The questionnaires were administered at the Occupational Therapy Unit, RIPAS Hospital and took 25-30 minutes to complete. Patients who had completed rehabilitation had the option of being seen at their residence to complete the questionnaires while patients who lived outside the main district were interviewed via telephone. For telephone interviews, the researcher ensured that the questions were addressed thoroughly and would restate the patient’s answer to make sure the correct answer was recorded.

For comparison, diabetic patients attending their follow-up appointments at the Diabetic Clinic in RIPAS Hospital were invited to participate in the study. A total of 50 patients were approached and 30 non-amputee patients were selected after cross-matching the age and gender with the diabetic amputees. They completed the SF-36 questionnaires and data was collected over a period of two weeks.

Data was analysed using RStudio version 1.2.1335 (2019) software for Windows 10. Independent t test was used to compare between below knee amputees and diabetic non-amputees while Wilcoxon rank-sum test compared below knee amputees with prosthesis and those without prosthesis. A p value of <0.05 was considered statistically significant.

This study was approved by the Medical and Health Research Ethics Committee, Ministry of Health, Brunei Darussalam and the Ethics Committee of PAPRSB Institute of Health Sciences (IHSREC), Universiti Brunei Darussalam (Reference No. UBD/PAPRSBIHSREC/2018/117).

## Results:

Of the 30 participants with TTA included in this study, 19 (63.3%) were males and 29 (96.7%) were Malay with a mean age of 57.4 years (SD= 7.43). 29 (96.7%) had diabetes, 22 (73.3%) had hypertension and 19 (63.3%) had hyperlipidaemia, whereas almost all had vascular problems related to diabetes and chronic kidney disease as the cause of their amputation. 18 (60%) of the amputees were fitted with prosthesis and 15 (50%) reported having phantom pain while 3 (10%) had problems with wound healing. Phantom pain was noted in 7 prosthesis users and 8 nonusers. The sociodemographic and amputation characteristics are presented in Table I.

**Table I:** Socio-demographic and amputation characteristics of TTA participants (n=30)

Study Variables	n	%	Mean (SD)	95% CI
**Socio-demographic Characteristics**				
Age (in years)			57.4 (7.43)	(54.63, 60.17)
Gender				
Male	19	63.3		
Female	11	33.3		
Ethnicity				
Malay	29	96.7		
Chinese	1	3.3		
Marital Status				
Single	1	3.3		
Married	29	96.7		
Education Level				
Primary	11	36.7		
Secondary	16	53.6		
Tertiary	3	10.0		
Employment Status				
Working	7	23.3		
Not Working	8	26.7		
Retired	15	50.0		
Comorbidities				
Diabetes	29	96. 7		
Hypertension	22	73.3		
Hyperlipidaemia	19	63.3		
Renal Disease	2	6.7		
Others ^a^	3	10.0		
**Amputation Characteristics**				
Causes				
Diabetes/Vascular	29	96.7		
Trauma	1	3.3		
Years since amputation			1.79 (0.91)	(1.44, 2.13)
Amputation Level				
Left TTA	12	40.0		
Right TTA	18	60.0		
Prosthesis				
Yes	18	60.0		
No	12	40.0		
Phantom pain				
Yes	15	50.0		
No	15	50.0		
Wound Healing				
Eventful	3	10.0		
Uneventful	27	90.0		

TTA= Transtibial Amputees; aIncludes Ischaemic Heart Disease, Retinopathy and Hemiparesis

Patients with TTA were found to have significantly lower quality of life scores for physical functioning, role limitations due to physical health problems, emotional well-being, social functioning and bodily pain, as measured by the SF-36 survey, than the diabetic non-amputees (Table II).

**Table II:** Comparison in QoL scores (SF-36) between TTA and Diabetic Non-TTA

Study Variables	n	Mean (SD)	Mean difference (95% CI)	t stat. (df)^a^	p value^a^
**Physical Functioning**					
Transtibial Amputees	30	34.5 (24.82)	48.0 (36.16,59.84)	8.11 (58)	<0.001*
Non-Amputees	30	82.5 (20.83)			
**Role limitations due to physical health**					
Transtibial Amputees	30	10.0 (20.34)	55.0 (37.18,72.82)	6.23 (41)^b^	<0.001*^b^
Non-Amputees	30	65.0 (43.84)			
**Role limitations due to emotional health**					
Transtibial Amputees	30	64.4 (39.09)	13.4 (-6.19,32.88)	1.37 (58)	0.177
Non-Amputees	30	77.8 (36.45)			
**Energy/Fatigue**					
Transtibial Amputees	30	67.2 (18.08)	6.6 (-2.46,15.79)	1.46 (58)	0.149
Non-Amputees	30	73.8 (17.20)			
**Emotional Well-Being**					
Transtibial Amputees	30	77.6 (18.03)	11.7 (3.84,19.63)	2.97 (58)	0.004*
Non-Amputees	30	89.3 (11.90)			
**Social Functioning**					
Transtibial Amputees	30	57.1 (18.03)	29.6 (17.73,41.44)	4.99 (58)	<0.001*
Non-Amputees	30	86.7 (11.90)			
**Pain**					
Transtibial Amputees	30	58.8 (24.84)	18.4 (6.30,30.36)	3.05 (58)	0.003*
Non-Amputees	30	77.2 (21.60)			
**General Health**					
Transtibial Amputees	30	70.8 (19.96)	6.5 (-2.79,15.79)	1.40 (58)	0.167
Non-Amputees	30	77.3 (15.74)			
**Health Change**					
Transtibial Amputees	30	60.0 (29.07)	-0.8 (-12.85, 11.18)	-0.14 (58)	0.890
Non-Amputees	30	59.2 (15.37)			

^a^Independent t test (equal variance assumed); ^b^Independent t test (equal variance not assumed)

Similarly, it was found that TTA participants without prosthesis had significantly lower quality of life scores for the physical functioning domain as measured by the SF-36, than TTA participants with prosthesis. The other domains assessed by the SF-36 were not significantly different, as shown in Table III.

**Table III:** Comparison in QoL scores (SF-36) between TTA with prosthesis and TTA without prosthesis

Study Variables	n	Mean (SD)	95% CI	Wilcoxon rank-sum test	p value
**Physical Functioning**					
TTA with prosthesis	18	44.4 (25.14)	(31.94, 56.95)	45.0	0.008*
TTA without prosthesis	12	19.6 (15.59)	(9.68, 29.49)		
**Role limitations due to physical health**					
TTA with prosthesis	18	11.1 (21.39)	(0.47, 21.75)	98.0	0.587
TTA without prosthesis	12	8.3 (19.46)	(-4.03, 20.70)		
**Role limitations due to emotional health**					
TTA with prosthesis	18	64.8 (38.73)	(45.54, 84.07)	106.5	0.963
TTA without prosthesis	12	63.9 (41.34)	(37.61, 90.15)		
**Energy/Fatigue**					
TTA with prosthesis	18	70.6 (11.74)	(64.72, 76.40)	94.0	0.561
TTA without prosthesis	12	62.1 (24.54)	(46.49, 77.67)		
**Emotional Well-Being**					
TTA with prosthesis	18	83.1 (12.00)	(77.14, 89.08)	66.5	0.081
TTA without prosthesis	12	69.3 (22.58)	(54.98, 83.68)		
**Social Functioning**					
TTA with prosthesis	18	59.7 (26.62)	(46.48, 72.96)	94.5	0.576
TTA without prosthesis	12	53.1 (29.25)	(34.54, 71.71)		
**Pain**					
TTA with prosthesis	18	59.2 (23.53)	(47.47, 70.87)	110.0	0.949
TTA without prosthesis	12	58.3 (27.76)	(40.69, 75.97)		
**General Health**					
TTA with prosthesis	18	75.0 (14.04)	(68.01, 81.98)	91.5	0.494
TTA without prosthesis	12	64.6 (25.98)	(40.69, 75.97)		
**Health Change**					
TTA with prosthesis	18	66.7 (24.25)	(54.61, 78.73)	77.0	0.150
TTA without prosthesis	12	50.0 (33.71)	(28.58, 71.42)		
TTA = Transtibial Amputees					

TTA = Transtibial Amputees

Eighteen participants with prosthesis in this study completed the Houghton Scale to assess their functional outcomes and their mean score was 4.89 (SD= 2.83). 55.6% reported wearing their prosthetic leg less than 25% of walking hours (1-3 hours) and required the use of one cane or stick for walking support. 83.3% felt unstable when walking with a prosthetic leg on slopes and rough ground as shown in Table IV.

**Table IV:** Prosthesis use in TTA with prosthesis

Houghton Scale (n=18)	n	%	Mean (SD)	95% CI
**Do you wear your prosthesis?**				
0. Less than 25% of walking hours (1-3 hours)	10	55.56		
1. Between 25% and 50% of walking hours (4-8 hours)	7	38.89		
2. More than 50% of walking hours (more than 8 hours)	1	5.56		
3. All working hours (12-16 hours)	0	0		
**Do you use your prosthesis to walk?**				
0. Just when visiting the doctor or limb fitting center	5	27.78		
1. At home but not to go outside	0			
2. Outside the home on occasion	5	27.78		
3. Inside and outside all the time	8	44.44		
**When going outside wearing your prosthesis, do you:**				
0. Use a wheel chair	3	16.67		
1. Use 2 crutches, 2 canes (sticks) or a walker	3	16.67		
2. Use one cane/stick	10	55.56		
3. Use nothing	2	11.11		
**When walking with prosthesis outside, do you feel unstable when:**				
a. Walking on a flat surface				
0. Yes	6	33.33		
1. No	12	66.67		
b. Walking on slopes				
0. Yes	15	83.33		
1. No	3	16.67		
c. Walking on rough ground				
0. Yes	15	83.33		
1. No	3	16.67		
H**oughton Scale Score**			4.89(2.83)	(3.48,6.29)

## Discussion

Our study showed that the quality of life in transtibial amputees was poorer in comparison to age and sex matched diabetic non-amputees affecting their physical functioning, role limitations, bodily pain, social functioning and emotional well-being. Phantom limb pain was reported in 50% of TTA patients. The use of prosthesis had a positive impact on the physical functioning for those with transtibial amputation. Poor functional outcomes and lack of prosthesis use were observed in TTA patients prescribed with prosthesis, and they still required supportive devices such as a cane or a stick to ambulate.

The mean age of subjects in our study is similar to those from Malaysia^8^ and Singapore^12^. Male preponderance was also a common feature. Almost all subjects in our study had vascular problems related to diabetes and chronic kidney disease as the cause of their amputation, similar to previous studies^8, 12^. Diabetic patients with complications have a higher risk for lower limb amputations^13^. Late presentations of foot lesions coupled with poor control of diabetes can accelerate the spread of infections and result in the need for amputation^14^.

Yusof *et al* compared the quality of life of diabetes amputees after major and minor while another study from Malaysia looking at the quality of life in lower limb amputees included 1/3 of patients with trauma as a cause for the amputation^4, 8^. Other studies looking at the quality of life in lower limb amputees had sample from India and Netherlands^7, 15^, both of these with trauma as the main indication for the amputation. A unique feature of our study is the comparison of quality of life in diabetic patients, with and without amputation.

Yusof *et al* used a similar assessment tool to the present study and reported better quality of life in minor amputees (at the level of ankle and below) compared to major amputees (at the level above the ankle including TTA)^8^. The subjects with minor amputation reported more pain and poorer social function. Razak *et al* used the WHOQOL-BREF questionnaire and found that psychosocial domain had influence on the quality of life while amputation affected the physical domain the most. The overall quality of life was found to be satisfactory^4^.

A study of 437 lower limb amputees done by van der Schans et al found a significant difference in the quality of life scores in terms of physical functioning and role limitations due to physical health and pain^15^. In the Indian sample, both physical component and mental component summary scores of the SF-36 were found to be significantly lower for amputees compared to the general population. Employment status, use of prosthesis or assistive device, stump and phantom pain were found to be predictors of quality of life in this study. Additionally comorbitidies were found to be an independent predictor of both physical and mental health component of QoL^7^.

The findings of our study are similar to the above mentioned studies. In addition, we observed lower QoL scores in the emotional well-being domain. This was also noted in a few other studies^7, 8^. A negative psychological impact of amputation in terms of depression, anxiety, sorrow and grief, distorted body image and psychosocial impairment has also been noted in the literature^16^. Losing a limb is a life-altering situation as not only locomotion is affected, but also the sense of self-confidence due to altered body image and social isolation. Therefore, effort should be made to restore the patient’s sense of control by providing emotional support to the patient and family, especially in the elderly as they are often worried about helplessness and dependency^14^. In addition, family and social support and understanding may play a role in the reintegration of TTA patients into society. In Brunei Darussalam, although efforts have been made to provide training and education for individuals with special needs, public places such as shopping complex and restaurants are still not fully equipped with facilities to accommodate the differently-abled, causing many of them to only stay at home. This further worsens social functioning and emotional health.

TTA participants in our study had more bodily pain compared to those without. This is similar to findings from other studies where subjects with major amputation had more bodily pain than those with minor amputation^8^ and amputees compared to the general population^7^. Apart from stump or phantom limb pain, other pain complications of post-amputation may include neuromas, reflex sympathetic dystrophy and bursitis or tendonitis^17^.

Half of the participants in our study experienced phantom limb pain. This was noted in both subjects using and not using the prosthesis. Immediate post-operative incidence of phantom pain and phantom sensation has been reported to be 72% and 84% respectively, while the incidence at six months post-operatively changes to 67% and 90%, respectively^18^. The mechanism of phantom limb pain and sensation remains unclear but is known to have a significant negative impact on the rehabilitation of an amputee^18^. Presence of phantom pain was also found to be one of the important determinants of health-related quality of life^2, 15^. It was seen to affect the physical component more than the mental component of QoL^7^.

Our study found that TTA participants without prosthesis had significantly lower quality of life scores for the physical functioning domain as measured by the SF-36, than TTA with prosthesis. This observation is similar to findings of other studies reporting the use of prosthesis having effect on the QoL^4, 7^. This suggests that a prosthetic limb helped to improve their physical functioning. However, other factors such as age, the presence of comorbidities and adherence to ambulation training might have an impact on physical functioning in TTA patients with prosthetic limbs. Patients with advanced vascular pathology may be less likely to use a prosthetic limb due to poor skin integrity, delayed healing, and impaired aerobic capacity/endurance, while other limiting factors include joint contractures and obesity^14^. Higher mortality and hence poor general health was evident in our sample with only 59 patients out of 107 who underwent TTA surviving at the time of the study. Even those who survived may be suffering from retinopathy, peripheral neuropathy with proprioception deficit, and needing dialysis every other day as well as need to attend multiple clinic appointments. Though not studied in detail in the present study, these factors may also impact the use of prosthesis as well as QoL.

The mean Houghton Scale Score for TTA patients fitted with prosthetic limb was 4.89, which corresponds to the K1 functional level (ability to walk on level, indoor surfaces) of the Medicare K-level classifications^11^. This suggests poor functional outcome and might contribute to the low quality of life in our participants. The causation may be age or comorbidity related, however, further investigations ought to consider the types of the artificial limb given and their comfortability as well. Choice of prosthetic device should be individualised based on the functional capacity and goals of the person, with the intent to allow the highest possible level of function^14^. About 55% of TTA participants with prosthesis reported wearing their prosthetic limb less than 25% of walking hours (1-3 hours) and would still require the use of one cane/stick for walking support. Majority (83%) felt unstable when walking with a prosthetic leg on slopes and rough ground. A possible explanation for this could be the use of prosthesis leading to alteration of patients’ movement biomechanics, proprioception, poor prosthetic fit and alignment, postural changes, and leg-length discrepancy^19^.

These possible reasons may explain why the participants do not use their prosthetic limb regularly and feel unstable when walking on rough grounds and slopes. Use of assistive devices may also suggest lack of confidence in the prosthesis, increase in limitations faced by the amputee, lack of infrastructure and social acceptance^7^. Exploration of reasons for not using prosthesis in the present study were inability to complete (recurrent admission for comorbidities) or defaulted rehabilitation (n=8) and patient found unsuitable for prosthesis use (n=4). However, further investigations will need to include factors such as the presence of visual and vestibular problems as they could also impact their ability to ambulate.

To try and identify areas for improvement in the light of findings of this study, it is important to review the process of rehabilitation of amputees in RIPAS Hospital. Access to rehabilitation has been noted to determine QoL in amputees^2^. In the preoperative stage, a referral is made to the Rehabilitation team lead by a Consultant in rehabilitation medicine. Other allied units include the Occupational therapy and Physiotherapy units. The rehabilitation medicine consultant reviews the general health of the patient, comorbidities and counsels the patient and family about the planned procedure. The physiotherapist advice on exercises for chest, upper limb and the unaffected limb and the occupational therapist conducts home assessment and activities of daily living. If necessary, patients are referred to the clinical psychologist who is not a regular part of the team. After adequate wound healing, the patient begins the preprosthetic rehabilitation phase where Pneumatic Post Amputation Mobility Aid is used with a target to achieve independent standing and walking for 25m and K1 functional level^16^. Once these targets are achieved, the patient is referred to the inhouse Orthotic and Prosthetic Unit for the fabrication of prosthesis. This unit is led by a qualified Orthotist and Prosthetist who also arranges follow-up for any ongoing problems related to the prosthesis. The prosthesis is provided free of cost to the citizens and permanent residents of Brunei Darussalam.

Fortington *et al* have shown that physical function in a lower limb amputee remains lower than the population norm about 18 months after the surgery. They reported improvement in the quality of life in the first six months^20^. The mean follow-up of about two years of the present study would suggest that the patient has reached their best possible outcome and it can be taken as the true reflection of their QoL.

Various limitations of the study must be noted. The sample size was small with high mortality in the cases undergoing TTA. The Malay translation of the Houghton Scale was not psychometrically tested and some of the patients included in the study had to be interviewed via telephone and not face-to-face, which may limit the validity of their answers. The data on QoL before the amputation was not available and moreover, the effect of various confounders on the QoL life has not been studied.

## Conclusion

The quality of life in transtibial amputees was poorer in comparison to age and sex-matched diabetic non-amputees. This was more pronounced in the domains of physical functioning, role limitations due to physical health, bodily pain, social functioning, and emotional well-being. The emotional health of transtibial amputees should be addressed during rehabilitation. The use of prosthesis only impacts the physical functioning in a patient with TTA. Further studies are needed to assess the predictors of poor QoL in the amputees and also explore reasons for patients reporting problems with prosthesis use. This would help in addressing the gaps in the management of transtibial amputees in Brunei Darussalam.
